# Observations on the Structure and Diseases of the Testis

**Published:** 1831-01-01

**Authors:** 


### Medico-Chirurgical Review,

### No. XXVII.

OCTOBER 1, 1830, to JANUARY 1, 1831.

### I.

Observations on the Structure and Diseases of the Testis. By *Sir Astley Cooper*, Bart., F.R.S. Serjeant-Surgeon to the King, Consulting Surgeon to Guy's Hospital, &c. &c. Royal 4to. pp. 245. 14 Plates. London, 1830.

[Second Analytical Article.]

Having fully treated in our last article on the simple diseases of the testicle, and on those which are specific but not malignant, it remains for us to consider the malignant affections of the organ, and the several diseases of its tunics. The study of the malignant diseases of the testicle is one of paramount importance, inasmuch as on our knowledge of the symptoms they present, depends our performance of an ugly, painful, and dangerous operation, with strict propriety and just necessity. The surgeon well acquainted with this interesting branch of his profession will operate where an ignorant practitioner would refuse, and refuse where the other would operate. It requires a powerful motive to induce a man of the slightest degree of sensibility, to contemplate without a sigh, or to study without a shudder these melancholy maladies. How painful a spectacle it must be to witness the march and the ravages of a disease, which is loathsome to the eye, and disgusting to the sense—to see a miserable fellow-creature worn down to the grave by a foul and a stinking cancer, bleeding, sloughing, spreading before his eyes, and eating from day to day and from hour to hour to the very core of his existence. Yet the poor wretch too often hugs hope to the last, and when all around wish him dead from very pity, he eyes his malady with solicitude and alarm, and implores from art a respite which it cannot afford.

### I. The Fungoid Disease.

This has been described by different authors under the terms of pulpy, medullary, soft cancer, and fungus hæmatodes. The term *fungus* is most applicable, for, when it ulcerates, it forms a large fungoid projection, full of blood, and bleeding from the slightest laceration, or from none at all. It has been designated medullary because it resembles brain; pulpy, because of its softness; and soft cancer, because it resembles scirrhus. Sir Astley, however, loves simplicity in surgery, and as a name is but a name, he confines the appellation of the disease to fungous or fungoid.

Its symptoms are as follow. It begins with an enlargement of the body of the testis, at first attended with even scirrhous hardness. The enlargement extends so rapidly, that in three or four months the whole body of the testis will be diseased, and then the epididymis becomes affected from one end to the other. Whilst confined to the testis the swelling is globular, but when the epididymis also is diseased it is pyriform, and so much resembles hydrocele as to be readily mistaken for it. This deception is increased by a small quantity of water being frequently effused, on which account the complaint has been denominated hydro-sarcocele. When carefully examined, the solid swelling is felt through the water, and the sides are found flatter than the fore part. The surface of the testicle is frequently uneven, but not in the early stages.

At first there is little pain, but the part is soon the seat of occasional darting pains, following the course of the spermatic cord to the groin and loins. If much handled it remains tender and more painful. The growth is uncertain; it sometimes increases quickly to a great size; at others, months may elapse before it is considerable. Neither is its growth steady and equal, but becoming very painful for two or three days it increases rapidly, and then remains stationary for two or three weeks. Slight causes will kindle it into activity; a catarrh, a little more exercise than usual will light up fresh inflammation. At first the scrotum is unchanged, and the cord is healthy; but the veins of the latter become enlarged.

The constitution seems little affected, but some of the secretions are found upon inquiry to be imperfect; the appetite is lessened, the body costive, the motions deficient in bile. Some disappointment, suspense, or anxiety has mostly been for some time in existence. Such are the symptoms of the first stage.

In the second, the scrotum is covered by varicose veins; the testis yields to pressure, so as to counterfeit the presence of water; the pyriform swelling gives force to the suspicion of hydrocele; and, as the water produced by the irritation of the disease increases, the mistake is still more readily made. The patient now complains of occasional darting pains in the tumour, tenderness of the testicle on pressure, and uneasiness in the back. The cord becomes thickened to the ring; the veins are enlarged; the countenance is sallow; in the centre of the cheek is a small fixed blush. There is sometimes constipation, at others diarrhœa, which relieves the symptoms for a time. The appetite is defective, the rest often disturbed by pain and irritation, the body becomes emaciated.

In the third stage the testis adheres to the scrotum, which no longer moves readily over it. One or more absorbent glands enlarge in the groin of the affected side, and if many are implicated, those on the opposite side suffer also. Opposite the adhesion of the scrotum the testis appears knotted and unequal on its surface, and similar inequalities exist in other parts of it. The cord grows enlarged, contracted,* hardened, varicose; sometimes it adheres to the pubes; the testis is firmly bound there.

The veins of the scrotum enlarge and a purple blush appears in one spot. The sense of fluctuation is then so distinct that the surgeon puts his lancet

---

* It is difficult to imagine enlargement and contraction simultaneously occurring. Perhaps compact would be a better term.—REV.

into it; he is surprised to see blood only issue from a spongy structure. The wound heals, but soon afterwards ulceration ensues, and a fungus projects, bleeds, discharges a gleety serum of peculiarly faint odour, spreads in two or three weeks to the breadth of the palm, sloughs often, is extremely offensive, occasionally very painful, not tender to the touch, and gives issue to a brain-like substance, if the testis be compressed. At length the miserable patient sinks from hæmorrhage, discharge, and constant irritation.

But ulceration does not always occur. Water in increased quantity forms in the tunica vaginalis. Most frequently the cord becomes enlarged and tuberculated, and leads to a tumour just below the kidney, distinctly felt if the thighs be bent and the abdominal muscles relaxed. The pain is acute; there is often colic; nausea, vomiting, great pain in the abdomen soon after swallowing food. The patient is attacked with diarrhœa—his appetite is gone—his countenance sallow and wan, and his eyes yellow—he grows very thin—the thighs and legs first swell on the diseased side and then upon both, accompanied by pricking pains—his abdomen enlarges—he has hiccough—rapid pulse—profuse perspirations—and, he dies. The duration of the struggle varies in different individuals. If the disease be malignant from the first, it has run its career in twelve months; if, at first simply chronic, it degenerates into malignancy, years may elapse before the fatal termination arrives.

*Dissection of the Fungoid Disease.* It is excessively hard in its commencement, and now, when examined by dissection, the effusion is found occupying only a part of the substance of the testicle. In a case, in which the disease had only existed four months, Sir Astley found that the hardness was not occasioned by the very solid nature of the substance effused, but by the excessive distention and unyielding nature of the albuginea. The effused substance was fibrous, of yellowish white colour tinged with blood, partially vascular, and, when macerated, flocculent, and looking like matted wool. The seminiferous tubes ceased to be observable at that part of the testis, but in others they remained entire.

In the second stage of the disease, a similar soft and white fibrous matter occupies the whole of the testis and epididymis, the parts of which readily yield to pressure. Intermixed with this specific deposite is yellow fibrine, or coagulated lymph, the result of common inflammation. When macerated in this state the soft specific effusion is removed, and leaves behind, like a cellular structure, the tendinous septa of the testis which enclosed it.

In the third and last stage, when the testis has been excessively enlarged, the tunica vaginalis contains a considerable quantity of water; the albuginea has given way; and a portion of the disease projects through it in the scrotum, giving rise to the inequality felt upon the surface of the testis, and the contamination of the absorbent glands in the groin. The interior of the testis contains cysts of serum, coagulated blood, and a white soft fibrous matter, which, when compressed, gives issue to a creamy substance, compared to putrid brain. If the scrotum has been ulcerated, then a fungus, of precisely the same material as the disease, projects from the interior of the testis. The epididymis is enlarged, and the tunica vaginalis adheres to the surface of the testicle where serum has not been effused.

The spermatic cord is excessively enlarged, indurated, and tuberculated, from the morbid secretion being irregularly deposited; but in some fatal cases the cord is not diseased. A quantity of serum is found in the abdomen, and behind the duodenum is placed a large tumour, adhering to that intestine in front, and concealing the aorta and vena cava behind. It varies in dimensions, from those of a clenched hand to the size of a child's head. When cut into, it contains a soft yet solid fibrine, intermixed with a creamy blood-stained fluid. In some persons the tumour begins from the lower part of the loins, and extends to the diaphragm, involving the kidney; on attempting to dissect it, a cream-like matter bursts from its various parts.

The aorta and cava are diseased, fungous tubercles and effusion being produced in their coats, and fungous effusion into the interior of the former. In many instances the mesenteric glands are affected. The omentum is sometimes thickened and puckered up. There are commonly tubercles in the liver, and Dr. Farre has engraved a drawing of the liver of a child thus diseased. In the collection at St. Thomas's Hospital is a preparation of the thoracic duct obliterated by this disease, and forming a tubercle at one part, as large as a walnut.

Sir Astley takes no notice of affection of the lungs in these cases, but Mr. Brodie has frequently seen patients die with symptoms of hydrothorax, after the removal of fungous testicles. On dissection effusion is found in the cavities of the pleuræ, and fungous tubercles in the lungs. We have ourselves seen fungoid deposites in the lungs, as well as in the cranial diploë and liver, after amputation of the breast for fungoid disease. There can be no question then, that the lungs are not unfrequently implicated in these cases, though the fact appears to have escaped Sir Astley's observation. We have also seen a case of fungous tumour in the neck after castration for this disease. The patient had another such tumour in the abdomen, and died of peritonitis occasioned by the latter. We have mentioned that, after amputation of the breast, several such tumours formed in the diploë of the cranial bones, and it is not improbable that the same may occur after amputation of the testis. The fact is, that these secondary malignant depositions may be formed in any texture of the body, though the lumbar glands appear to be most frequently affected, when the testicle has been diseased. Of the viscera the liver is most commonly concerned.

The nature of the disease is, like that of every morbid growth, enveloped in obscurity, but yet the more palpable characters are not beyond our ken. It differs from common inflammation, says our able author, in soft fibrine being effused, instead of the healthy and solid albumen of the blood. Its texture allows of organization in some parts, but in others it is too weak to support the blood-vessels shooting into it, and the blood is extravasated in consequence. In some parts serum is effused, and cysts are formed which contain a fungus in the interior. When injection is thrown in by the arteries and sections made of the disease, some parts are coloured, whilst others are entirely unorganized. When injection is thrown in by the veins they are found very large and varicose.

If the fungus produced by the disease be carefully examined, some parts bleed from the slightest touch, in consequence of the frailness of the coats of the vessels, and others slough, from the want of organization. The surrounding arteries are *not* much enlarged, but the vessels of the disease have no contractility, and very tender coats; they bleed profusely when cut into.

The best mode of examination is to inject the part and macerate it; the soft and thickened fibrine are separated, and the supporting tissue will appear.

*Cause of the Disease.* It is local or constitutional. It is sometimes founded in a constitution naturally feeble. Sir Astley has seen it in infancy, and the child had greatly the scrofulous character, and even in some cases where the patient was adult, he has thought the same diathesis prevailed. It has been remarked, we believe, that fungus hæmatodes of the eye very frequently occurs in scrofulous children, and in most of the cases of fungus hæmatodes in other parts which we have witnessed, the patient has appeared to us to be of strumous habit. But perhaps this goes for little, as scrofulous persons are more disposed than others to all diseases, from their natural delicacy and weakness of conformation.

It occurs also in those who have been originally healthy, but whose constitutions have been broken down by anxiety of mind, intemperance, over exertions mental and corporeal, and want of attention to the due performance of the secretions. In such persons a slight feverish state is induced; the tongue is white with a yellow streak in its middle; the appetite and digestion are defective, probably from depraved gastric secretion; the bowels are costive; the bile absorbed, and the eye consequently yellow; the pulse frequent; the cheek flushed, though the skin is sallow; the nervous system irritable; the rest broken. In such a state of constitution a slight injury gives rise to an unhealthy local action, and a peculiar and unnatural secretion is the consequence. When the local disease has existed for some time, the absorbents become irritated, and the diseased action is extended to their glands. Other structures then become irritated also; similar diseases occur out of the line of absorbent irritation; and then various parts of the body are attacked, for the same constitution will produce the same local action, under accidental but continued irritation.

As a proof that the disease is constitutional, we find that there exists a disposition to form it in various portions of the body; and that there is also a peculiarity in the local action, is shewn by the wound for the extirpation of the part often healing in the kindliest manner, the disease, notwithstanding, recurring in the cord or elsewhere. This proves that the local action differs from common inflammation, and when the disease returns, it is after common inflammation has ceased. The state of the blood also favours the production of the disease; for occasionally, when drawn, it coagulates very weakly, and the serum is large in quantity, and of a deep yellow colour.

Such are the opinions of Sir Astley Cooper on the nature and causes of fungoid disease. If they add little to our precise knowledge of this mysterious subject, it is only what has happened with the speculations of the ablest of his predecessors. We know, indeed, little of the laws which influence morbid growths, and we fear that we are not likely to know much. It is clear that the fungoid disease, if not constitutional at its onset, very soon becomes so, and that little is effected by the knife, even when resorted to under the most favourable circumstances. This is at present the most practical view we can take of the subject.

*Diagnosis.* "From hydrocele it is often difficultto distinguish it, if in hydrocele the fluid be opaque, or the tunica vaginalis be greatly thickened; but in a very large proportion of cases hydrocele is transparent, and therefore it is only required to press the fluid to the fore-part of the tunica vaginalis, and to render the scrotum very tense, and the fluid is seen, either by the sun's rays falling through the part, or by the light of a candle in a room otherwise darkened. Besides, this disease, in its second and third stages, yields rather than fluctuates; but still the distinction is sometimes difficult, and all candid persons will confess they have erred, and when they have believed it water, have found it solid. Mr. Pott, Mr. Hunter, Mr. Cline, and many others have been thus deceived; and I am ready to confess that I have more than once been mistaken." 128.

In all cases of doubt Sir Astley advises us to make a small incision in the scrotum, and then puncture the tunica vaginalis; the tunica vaginalis should always be opened before castration. From the hydatid testis, the fungoid disease is only distinguishable by the health remaining perfect in the former. As each disease requires amputation, the diagnosis is less important. The excessive hardness of fungus in the first stage, and its yielding or obscure fluctuation in the second, distinguish it from chronic enlargement of the testis. We now arrive at the

*Treatment.* When once this horrible disease is formed, we know of no treatment that can cure it. Improvement of the health and diminution of local increased action may retard its progress, but they do no more. Sir Astley suggests the trial of the various new medicines. Plummer's pill, sarsaparilla, leeches, have been advised; but why should we persevere with what have often failed? We possess then nothing that will cure the disease, but it is right to give medicines that will alter and improve the constitution, prior and subsequent to an operation. Calomel and opium in considerable doses at night, and inf. gent. c. with soda and rhubarb twice a-day, or the inf. calumb. instead of the gentian, are the medicines recommended by Sir Astley for this purpose. Evaporating lotions and the frequent use of leeches are the best local means.

But so soon as it is certain that the disease is of fungous character, no time should be wasted in exhibiting medicines. The operation should be performed immediately, and the constitutional remedies may be employed for several weeks after it. An early operation gives the only hope, and that is but a faint one; for in one case, in which it was performed in four months after the commencement of the disease, it was unsuccessful. Sir Astley is of opinion that it should not be delayed beyond three months, mercury having been previously tried.

He advises that the treatment recommended for the simple chronic disease be first fairly tried, and if it fail, no other that he knows of will succeed. The operation should be performed so soon as the patient has recovered from the effects of the mercury, for erysipelas is apt to follow if done immediately after the use of the mineral. If the testis has been suffered to grow large—if the cord be diseased, apparently only to the ring—if the appetite be impaired, with occasional vomiting—if there be occasional severe pain in the abdomen, tenderness on pressure, diarrhœa at one time and costiveness at another—under these circumstances, although no tumour can be felt, the disease will return in some other part of the body after operation. Sir Astley thinks he has seen one medicine of service, though doubt may reasonably be entertained upon the subject—it is the oxymur. hyd. given to gentle affection of the mouth, and continued for a length of time, in combination with the tinctures of bark and rhubarb, or the concentrated decoction of sarsaparilla. If the operation be performed, the surgeon must still attempt to invigorate and alter the constitution.

Such are Sir Astley's very practical statements, respecting the causes, nature, and treatment of the fungoid disease of the testicle. The prognosis to be drawn from them is gloomy, but certainly not more so than experienced surgeons will own to be strictly true. We do hear from time to time of fungous disease cured by medicines or by operations, when circumstances appeared most unpropitious. But the reporter will be found, on close examination, to have deluded others or to have been deceived himself. Let no such facts be trusted. In every point of view, it is better to err on the side of firm and rational scepticism, than on that of too easy belief and too general credulity. If this rule were implanted in the breasts of professional men, perchance we should be less encumbered with false facts, or, at all events, they would exert a less baneful influence on the precepts and practice of our art.

We have one remark to make on the exhibition of mercury in these cases. As our readers will have seen, it is given as in some degree the test of the benignity or malignancy of the disease. If it answers it is well. But if it fails we are not where we were before its exhibition, and that is the point to which we would direct attention. In two cases of the early stage of the fungoid disease, in which the diagnosis was of course doubtful, it was given with the same views as those advocated by Sir Astley. It disagreed in a marked manner. In one patient, more particularly, the health, which had previously been good, broke down; the disease increased suddenly; and after castration it shewed itself in the loins, with a rapidity unequalled by all that we have witnessed before or since.

We have also reason to believe that the lumbar glands are too often contaminated in the earlier stages of this and the hydatid disease, when no indication of this formidable feature is given by the symptoms of the case. Mr. Brodie amputated an hydatid testis in an early stage, the cord, if we remember right, being healthy. The man died of peritonitis, and the lumbar glands were found affected.* Mr. Hawkins lately amputated a testis for the fungous disease, in a tolerably early stage. The cord was a little enlarged, but so slightly that it continued for some time a question, whether it was enlarged at all. This patient also died of peritonitis, and the lumbar glands were found distempered.

Our able author details four cases of the disease. We shall give the particulars of two in this place, the remainder will be found in the Periscope department.

*Case* 1. James Watson, ætat. 40, was admitted into Guy's Hospital, for fungous disease of the left testis, which was extirpated. The wound healed kindly and the patient was discharged in the following month. In the course of ten weeks he was attacked with severe shooting and cutting pains,

* We have adverted to this case in the chapter on the hydatid disease.—*Rev.*

commencing in the part of the cord from which the testis was separated, and extending into the groin and loins, but occurring only once in a week or ten days. He was again admitted into the Hospital. The divided end of the cord was enlarged, hard, and painful upon pressure. The scrotum was not adherent. The tumour in the inguinal canal enlarged, and in two months he complained of pain in the abdomen on pressure. A large tumour was felt on the lower part of the left side of the sigmoid flexure of the colon, and in the region of the kidney. Another large tumour was also felt, extending towards the diaphragm. They were painful, especially on pressure, increased in size, emaciation advanced, constant and severe diarrhœa was established, and the patient died exhausted by continued irritation.

*Sectio Cadaveris.* On dividing the left side of the scrotum, a soft, white, pulpy tumour, as large as a walnut, was seen at the end of the remaining part of the spermatic cord. The latter was thickened, and another tumour of the same description as the first adhered to the peritoneum at the lower part of the abdomen. An immense tumour stretched up from the edge of the pelvis nearly to the diaphragm, filling almost the cavity of the abdomen. The large intestine passed over it, it enveloped the aorta and vena cava, and the left kidney was so implicated that its functions had been in all probability destroyed. When cut into or torn, a curd-like matter, resembling very thick cream tinged with blood, escaped from the openings. Many of the mesenteric glands were enlarged; the liver had two small white tubercles in its substance; the thoracic duct was healthy, but a small gland, apparently affected with the disease, was attached to it. The thoracic organs were healthy.

*Case* 2. This is communicated to our author by Mr. Lunn of Rotherham. A man, aged 48, a *Borer* by trade, applied to Mr. Lunn in March, 1809, for disease of the right testicle. There was great pain in the loins and now and then in the body of the testis, the enlargement of which resembled a hydrocele in all except its transparency. The scrotum was stretched, and a fine vascular net-work existed on its surface. Mr. Lunn made a puncture with a lancet and discharged about six ozs. of colourless serum. The testis was now ascertained to be about four inches long, and six or seven in circumference, not painful on moderate pressure. The spermatic cord presented a varicose enlargement of the veins; the vas deferens was perfectly sound. Mr. Lunn recommended extirpation, but the patient desired some milder remedy and resumed his work. Cicuta, mercury, and leeches were employed for a month, when the patient was much as before, but with increased pain, both in the testis and the back. The puncture was repeated, and nearly a pint of fluid discharged. The testis had increased in size and sensibility, and was somewhat uneven on its surface. The cord was as before.

On the 30th April the testicle was amputated in the usual way, and in eighteen days the patient was quite well. So he continued till August 1810, when he complained of violent pain in the back, which was removed by a purge of calomel and scammony. In September he relapsed and the untoward symptoms gradually increased. He complained of great weakness and numbness of the right leg, attended with œdematous swelling of the limb and a smarting pain at the cicatrix. In a few weeks the left leg became swelled and painful, and afterwards the cellular membrane about the nates, abdomen and chest, the penis and scrotum, was distended with a preternatural quantity of fluid, very distressing to the patient. The anus became nearly closed, and stools could only be procured by cathartics. At last, worn out with emaciation and excessive pain, he died in February, 1811, having survived the operation one year and ten months.

*Dissection.* On making an incision through the thickened abdominal parietes, several pints of brown serum escaped. The omentum was corrugated, contracted, hard, and knotty. The large intestines were universally thickened in their coats, with a spotted inflammatory appearance, and firm adhesions to each other and the neighbouring parts. The cæcum was united to and buried in the enlarged iliac glands on the right side, and the glands on the left were likewise much enlarged, and in a state of suppuration. The sigmoid flexure of the colon formed, along with the lumbar glands, a heterogeneous diseased mass, running into the pelvis, about the length and circumference of a man's fore-arm, and containing a great quantity of pus and curdy matter. The rectum was amalgamated with this horrid mass of disease, every gland within the pelvis partaking more or less of it. The mesenteric glands were of all sizes and in all stages of the disease. The pelvis was frequently filled with matter from the incisions; some of the glands cut like cream cheese. The lumbar glands were astonishingly large and contained about a pint of pus and curdy matter. The contents of all the glands were peculiarly white. No vestige of the cord on the right side was found.

It is impossible not to recognize in the account of this dissection, the ravages of fungus hæmatodes.

### II. THE SCIRRHOUS DISEASE OF THE TESTICLE.

Sir Astley doubts the existence of this disease in the form it assumes in the mamma: an excessively hard swelling, intersected by a net-work of strong bands or fibres.

"I have seen a few instances of a very solid enlargement of the testis, accompanied with great weight, attended with occasional pain, beginning in the body of the testis, never becoming soft, like fungus, or producing a fungoid and very vascular bleeding surface, but feeling tuberculated, irregular, and excessively hard, but never becoming so large as the fungoid diseases: the pain extending to the loins; the spermatic cord enlarged, hardened, and tuberculated; a smaller tumour than that of the fungoid disease forming in the abdomen; some water is secreted into the tunica vaginalis; at length a dropsical effusion into the cellular membrane of the leg and thigh of the diseased side is produced, and then the other leg becomes similarly affected. Ulceration I have once seen occur:—the testis gradually wasted under it—the glands of the groin became diseased—and the man, after some months, died. But this state rarely happens; for generally, without ulceration, the patient's countenance becomes sallow, and he sinks under impaired digestion, pain and tumour in the abdomen, and an irregular state of bowels, which are frequently accompanied with ascites." 151.

*Dissection of the Disease.* Water is found in the tunica vaginalis, so that there is hydro-sarcocele, as the old surgeons termed it. The tunica vaginalis is in parts adherent to the testis. In the testis a hard white mass, in lobes or tubercles, and little vascular, usurps the place of the seminiferous tubes; it is sometimes interspersed with small portions of cartilage or bone. The epididymis contains the same fibrous secretion; the cord is enlarged and has small white tubercles in it. The tumour in the abdomen is of white solid texture, very unlike that of the fungoid disease.

*Diagnosis.* The complaint is distinguished by its slow progress, its great hardness from first to last, its weight, and its irregular tuberculated feel. Like the fungoid, it is founded in a diseased state of the constitution and in a peculiar local action; like it, also, it extends by absorbent irritation. However, it occurs less in different parts of the body at the same time, and its fatal progress is less rapid. In scirrhus the secreted material is a solid compact fibrine, becoming vascular with difficulty, and its arteries always being small, though its veins are varicose. In the fungoid disease it is a soft fibrous mass, easily rendered vascular; bleeding readily; growing rapidly in some parts; too soft to support vessels in others; and then extravasations of blood take place.

These distinctions are laid down with much perspicuity. But they are not in all cases so clearly made out, neither by symptoms, nor afterwards by dissection. We have seen a tumour in the breast which, before amputation, resembled scirrhus in its hardness and fungus hæmatodes in its ulceration. When dissected, it was difficult to say to which morbid structure it belonged. Scirrhus and fungus-hæmatodes, then, occasionally run into and blend with each other, though commonly the broad and perceptible differences, pointed out by Sir Astley Cooper, are observed.

*Treatment.* Medicine appears to have no more power over this, than over the fungoid disease. The same course and plan must be adopted. Calomel and opium with leeches, evaporating lotions, and the recumbent posture, should be tried, and if they fail the remainder is confessedly experimental. We have more time allowed us, and that is a great point, but we must not delay castration so long as to endanger disease in the cord or a tumour in the abdomen. In the local treatment of the fungoid and scirrhous inflammation, our object should be to occasion sloughing of the sores, when they have gone into ulceration. Our author has seen a very large fungous tumour of the breast slough away; the wound healed, the woman was discharged well, and she did not return. The result is of course uncertain. We have seen a fungous tumour of the breast slough deeply, and the remainder of the gland was removed by the knife. The patient left St. George's Hospital with the wound healed, but in two or three months she died with fungous tumours in the diploë of the cranium, in the lungs, and liver. We have referred to this case already.

Extensive sloughs of the fungoid projection may be produced by powdered alum, sprinkled on its surface. To cleanse the sore and remove the offensive smell, the nitric acid, in the proportion of ʒj. to ℔ij. of distilled water, is an excellent application. The chlorates of lime and soda are also useful in the same point of view. We have seen the ioduret of mercury, much lauded by Biett in cases of lupus, tried on a cancerous ulcer of the breast; it irritated and was discontinued. We would say of the chlorates, from what we have seen of their use, that they are cleanly applications, useful in foul-looking sores, but certainly not adapted to cases of decided sloughing. For that, the balsams and more energetic applications are required. Sir Astley only details one case of the scirrhous disease of the testis.

*Case.* Thomas Cheston, aged 44, was admitted into Guy's Hospital, with enlargement and induration of the testis and epididymis. The spermatic cord was not enlarged. He had much pain in the loins, increased on stooping—his countenance was sallow—his digestion impaired—his leg and thigh œdematous. He had been a strong muscular man, and thought he was in good health when the disease began, which was in June, 1808. He first observed pain in the loins, with hardness and uneasiness in the testicle, which slowly increased in size but never became large. Water formed around the testis, and, when four ounces were drawn off, the indurated mass was felt through the surrounding fluid.

The testis was removed in March, 1809, and the wound slowly healed. He was then discharged from the hospital, the swelling still remaining in the leg and thigh, and in a month after his return to Tottenham he died. On examination of the testicle after its removal it was found hard, white, compact, tuberculated, and in a few spots very vascular. The epididymis w asalso enlarged.

This concludes the account of the diseases of the testicle and epididymis. Before proceeding to the affections of the tunics, Sir Astley has a chapter on the operation of castration which deserves attention.

### III. ON THE OPERATION OF CASTRATION.

This operation is required for the chronic inflammation of the testis, when it has ulcerated and formed a granular swelling, by which a large proportion of the testis protrudes. Sir Astley has also known castration required in chronic suppuration, when numerous sinuses have formed through the scrotum. For the irritable testis it is sometimes required, when medicine has proved abortive, and suffering renders existence burdensome. In these cases the operation is a straight-forward proceeding. But when it is contemplated for fungoid disease, it requires great judgment to determine when it shall be done, or whether it ought to be done at all. The disease when first dicovered may have long existed, and the surgeon will therefore inquire into its size at the time it was first perceived. He will also try to learn the patient's previous health, and ascertain if he has suffered from pain in the loins, or severe dyspeptic symptoms; for disease in the testis is sometimes not merely the precursor but concomitant of disease in the abdomen.

*Case.* Mr. ———, æt. 52, has now enlargement of the right testis to five times, and, of the left, to three times their natural size. There is water in the tunica vaginalis on each side. The scrotum is painful, and its veins are enlarged and numerous. The spermatic cord on the right side is enlarged, and a tumour can be felt in the course of the spermatic vessels, below and before the right kidney. The liver and loin on that side are extremely tender on pressure. The right leg has been swelled for a week. He has quick pulse; slight but constant fever; great thirst; occasional shortness of breath, particularly after taking food; debility; emaciation; and anxious countenance.

In the early part of 1829 he had an attack of hemiplegia, succeeded by severe lumbago. In September of the same year he found the right testicle enlarged, hardened, and surrounded with water; and in a few days afterwards the left testis became affected in a similar way. He was frequently sick, and had occasionally severe pain in the abdomen. He had suffered greatly from anxiety of mind.

In the appendix we learn the issue of this case. In September, 1829, the tunica vaginalis was punctured by Mr. Callaway, and the fluid evacuated; it immediately coagulated. In November, he was attacked with daily paroxysms of pyrexia, ushered in by a rigor and terminating with perspiration. In December he was seized with a slight epileptic attack, or rather we should say from the symptoms, apoplectic. From this he soon recovered, but that day was to him as a blank in his existence. He had now daily paroxysms of fever, and several spasmodic attacks in the muscles of the legs and arms. His health gave way; he frequently complained of shooting pains in the head, occasionally accompanied with slight delirium and double vision. At times this was followed by copious perspirations. In February, 1830, he died. For three weeks previous to his death, the bowels rarely acted without an enema. This poor gentleman was a surgeon.

*Dissection.* The left testis was greatly enlarged, and a large hydrocele existed in the tunica vaginalis. When the testis was cut open it was found to be loaded internally with a soft secretion, which did not seem vascular, and in many parts was semi-fluid. The cord was free from disease. The absorbent glands in the course of the spermatic vessels was slightly enlarged, and had a white appearance internally. The right testis was enlarged and contained a white solid matter; there was hydrocele also. The cord was not diseased, but the absorbent glands were affected like those on the opposite side. The vesiculæ seminales, prostate, and lower part of the vasa deferentia behind the bladder, were distended with a similar white substance and greatly enlarged. The substance in question was unorganized. The internal iliac and hypogastric arteries on both sides were obliterated in parts by a hæmatoid substance, which adhered very strongly and inseparably to their coats. The colon, near the cæcum, presented a stricture with thickening, and a circular ulceration of the mucous membrane of malignant character. The left kidney contained a fungoid tubercle, of a darker colour than that in the testis. The ureter was enlarged. The right kidney was wasted to a third of its natural size, and its pelvis and ureter were excessively enlarged to the termination of the latter in the bladder. The lungs were tuberculated and ulcerated.

Sir Astley observes that in this case there was a mixture of scrofulous and fungoid disease. In such an individual, the disease in the testis is the result of a constitution broken down by anxiety of mind, and the concomitant of worse abdominal complaints. If the patient did not die, as he probably would, of an operation, it would only deprive him of a part of his disease, which would certainly proceed with more inveterate malignancy elsewhere.

Before attempting the operation it is necessary to examine minutely the state of the cord, to learn if it be diseased to the abdominal ring. If it is, castration ought not to be performed, for, Sir Astley has never known it succeed. If any of the glands in the groin are affected, the operation is also forbidden. Sir Astley confesses that the fungoid disease is the most malignant to which the body is exposed; it is uncontrollable by medicine, rarely successfully removed by operation.

In the scirrhous disease, we have not only more time allowed for the trial of medicine and the performance of the operation, but the success of the latter is somewhat greater. When the cord is diseased, the same objections lie against castration, as in the fungoid affection of the testis. In a case of hardened and enlarged testis, with the cord greatly thickened and indurated to the abdominal ring, Sir Astley refused to operate, deeming such a step improper. A pupil at the hospital took lodgings for the man in the country, and removed the testis. The patient died of peritonitis. This ignorant head-strong fool deserved a severer punishment for his conduct than his patient's death.

We now arrive at the mode of performing the operation, recommended and practised by our excellent author.

The pubes being clearly shaved on either side, a table of convenient height is prepared by covering it with two blankets and a sheet; a T bandage is fastened round the patient's loins.

A small incision is then made into the tunica vaginalis on the fore part of the testis, to satisfy the surgeon that the disease is neither hydrocele nor hæmatocele. This being determined, the operation is begun by *first* making an incision from the abdominal ring to the very lowest part of the scrotum. From this, two advantages arise;—first, matter is prevented from accumulating in the scrotum, when the suppurative inflammation begins: and secondly, the testis is much more easily removed. In the next place the fascial sheath of the spermatic cord is to be opened below the abdominal ring, and the cord to be completely exposed. Thirdly, the cord is to be well pinched up between the fingers, and a tenaculum, or needle and ligature should be thrust through it and given to an assistant, to prevent its retraction. Sir Astley has always done this since Mr. Cline informed him that he once saw the cord retract within the inguinal canal, occasioning some bleeding and considerable perplexity. The fourth step is to cut through the cord. Sir Astley protests strongly against dissecting round the testis before the cord is divided, a proceeding which lengthens the time of the operation, and adds materially to the sufferings of the patient. Fifthly, the lower portion of the cord is to be taken hold of by the surgeon, and by it the testis is to be drawn from the scrotum, its adhesions being cut as it is drawn out. This plan is in general easily executed, and if there be adhesions to the scrotum, they are more readily divided by this than by any other method. Sixthly, lift up the cord with the tenaculum or ligature, and secure the spermatic artery with a fine ligature. Seventhly, turn the cord upwards towards the abdomen and see and secure the artery of the vas deferens, a vessel which frequently is not tied, and affords a most teazing and continued bleeding.

Sir Astley reprobates in the strongest manner, the not-ancient practice of tying the whole of the cord in one ligature. Mr. Chandler did this in 1807. The man complained at the time dreadfully—on the eighth day the ligature separated—on the following day tetanus began, and in three days afterwards he died.

Eighthly, secure the external pudic artery, which is often divided in making the upper part of the incision. If it bleed freely during the operation, let the assistant compress it with his finger. Ninthly, secure every vessel of the scrotum which continues to bleed, or which has been observed to bleed freely during the operation. Tenthly, make two sutures, at least, in the scrotum; and if the testis has been very large, or has adhered to it, a portion of the scrotum may be removed, to prevent its forming a loose bag for blood and pus.

The patient is to be carried to bed in a horizontal posture, without any dressings being placed on the wound. When all danger of bleeding has ceased, and not before, lint and adhesive plaster are applied, and the T bandage is to be secured. The patient is to be kept cool and covered only with a sheet; in Summer cold spirituous lotion should be kept upon the scrotum. The sutures should be removed in eight days, and the wound commonly heals in three weeks. Sir Astley once removed a diseased testis accompanied with hernia; he first reduced the hernia, and then dissected the cord from behind the sac. The patient had a chronic complaint in the testis; he did well. Sir Astley also removed, in Guy's Hospital, a diseased testis, accompanied by adhering omental hernia. The patient had no unfavourable symptoms.

Sir Astley makes no mention of the bad symptoms which occasionally follow castration. There is always some risk of suppuration and even sloughing in the loose cellular membrane of the scrotum. That we have seen. There is a disposition to secondary hæmorrhage, but this may be diminished, if not prevented, by carefully tying every vessel at the time, and observing Sir Astley's directions with regard to the management of the blood-vessels. If oozing of blood takes place, notwithstanding these precautions, then the application of cold to the scrotum is generally successful. The most efficient kind of cold is the application of pounded ice in a bladder, but although it arrests the hæmorrhage we think we have seen it in several instances productive of equivocal results. Men laugh now-a-days at the doctrine of revulsion, but very powerful repellents, and ice is one, are occasionally dangerous in spite of the sceptics. We have seen two instances of fatal peritonitis after castration, and Sir Astley details another. It is remarkable that in all the three the cord was either enlarged or the lumbar glands diseased. We have also seen a temporary retention or partial suppression of urine produced by the operation, and this is alluded to by Petit. With these few remarks we take leave of the operation of castration. We could have wished that Sir Astley had pointed out, from his wide experience and accurate observation, the usual immediate consequences of the operation which have been witnessed by himself. We are thankul for what we have got; it is impossible to feel dissatisfaction at the able and worthy Baronet.

### IV. On Hydrocele.

The tunica vaginalis is a serous membrane, and like all structures of that class, it is liable to dropsical effusion. When opened in its natural and living state, a halitus only arises from it, and then the surface becomes dry; but if from any cause there is a greater determination of blood to the part, the secretion becomes a fluid, which accumulates in considerable quantity and produces the disease called hydrocele. Hydrocele then is an accumulation of fluid in the tunica vaginalis, producing a pyriform, fluctuating, and generally transparent swelling in the scrotum. The term applies to any watery tumour, but is now limited to hydrocele of the tunica vaginalis and hydrocele of the spermatic cord.

*Symptoms of the Disease.* It begins opposite the lower part of the testis. It is unattended by pain and usually first discovered by accident; the fingers sinking readily through it when compressed, and the testis being distinctly perceived. As it increases, the swelling becomes tense and conceals the testis. It next assumes a pyriform shape, the broadest part below, and continues free from pain, till it acquires a great size, when its weight and tension occasion an uneasy sensation in the lower part of the back. Some few of the vessels of the scrotum are enlarged, but the skin is not inflamed. The general health is unaffected.

"When the swelling is attentively examined, it is generally found to be transparent; and as some Surgeons deny the truth of this opinion, it must arise from their not understanding the mode of examination. The room is to be darkened from the light of day: the patient holds a candle burning close to the side of the scrotum; and the Surgeon grasps the posterior part of the swelling, so as to render its fore-part as tense as possible; then the Surgeon, looking at the swelling from the opposite side to the candle, and placing his left hand on the fore-part of the scrotum, generally discovers its transparency. I have seen some Surgeons place a candle on one side, raise the scrotum, and look from the other, and say the swelling is not transparent:—no, and I should wonder if it were! for in this way it scarcely ever can be. The strong light of the sun falling directly upon the part, answers equally well in shewing its transparency, if the scrotum be rendered tense." 167.

Hydrocele has a distinct fluctuation, but when excessively distended feels hard. The testis is generally placed at the posterior part of the scrotum and two-thirds of the swelling downwards. Pressure there gives the sensation of squeezing the testis, and when the swelling is otherwise transparent, the testis may be seen there. Hydrocele is a very loose and moveable swelling; if it do not greatly distend the part in the course of the cord it bends easily on the abdomen, and moves readily in all directions.

Such is the usual character of the disease. Sometimes it is the result of inflammation of the testis, and then it is accompanied with pain, hardness, and swelling of the part, assuming more the form of the testis itself, and being less distinctly transparent. The fluid of hydrocele is like serum—yellow; transparent; coagulable by heat, acids, and alcohol; coagulating in port wine and in solutions of sulphate of zinc.

*Varieties of Hydrocele.* These are many. It sometimes exists on both sides of the scrotum; the swellings should be cured in succession. The testis varies in its situation. It is sometimes glued to the fore part of the tunica vaginalis, and the serum is accumulated on each side of it. A gentleman with this variety of hydrocele applied to a surgeon, who tapped it with a trocar, and, as no water followed, said, 'he was mistaken, and the testis must be removed.' The patient applied to another surgeon, who observing venereal spots on the abdomen, and a node upon the tibia, gave him mercury, with the effect of dispersing these symptoms, as well as the enlargement and hardness of the testicle. The nature of the hydrocele was now apparent; it was injected from the side, instead of the fore part; and the patient perfectly recovered.

Inflammation of the testis is often followed by partial adhesions of the tunica vaginalis; and then if water is effused, it is variously situated, of course, with respect to the testis. It is rarely found posteriorly, and when there, it is in consequence of the tunica vaginalis yielding to pressure; thus a pouch is produced, with rather a narrow orifice of communication with the tunica vaginalis. Sometimes the water is collected in distinct and separate cysts in the tunica vaginalis. A cyst is sometimes formed from the extremity of the epididymis, and hangs within the tunica vaginalis; this is generally accompanied by common hydrocele. Hydrocele sometimes forms two swellings with an hour-glass contraction between them: one is placed opposite the testicle, the other reaches to the ring, and the two have a smaller swelling of communication. This swelling reaches to the abdominal muscles, and, like hernia, dilates upon coughing. It is distinguished from it by its permanence, its transparency, its fluctuation, its history, and the absence of intestinal interruption. The latter diagnostic sign is, however, in all such cases to be received with this understanding:—that intestinal symptoms may be accidentally conjoined with this or any other swelling of the kind, and then the surgeon must depend on the other diagnostic marks.

Two distinct hydroceles sometimes exist on the same side, of which the following case offers an example.

*Case.* Mr. Roberts tapped a patient for hydrocele, but still there remained a swelling, not admitting of being emptied through the canula, and half as large as that which existed before. The patient came to London. Sir Astley tapped the lower hydrocele, and a yellow serous fluid was discharged, but still the other swelling was there. It extended from the upper part of the testis to the ring, and was very transparent. Sir Astley tapped it and drew off a watery fluid, free from colour, and containing less coagulable matter than common serum. He afterwards injected the lower hydrocele and repeatedly tapped the upper swelling. He believes it to have been encysted hydrocele of the cord, for in a shut hernial sac he has never discovered fluid, although he has examined three or four in the dead body.

As the communication of the tunica vaginalis with the abdomen does not always close at birth, but sometimes remains through life, water formed in the one passes into the other, and thus there is a hydrocele communicating with the cavity of the abdomen. Sir Astley has several times seen this in children, and occasionally also in the adult. In the case of a young gentleman with this affection, Sir Astley applied a truss to obliterate the communication, and it cured the hydrocele, which was absorbed. In this case, the water probably formed in the belly and descended into the tunica vaginalis. When hydrocele communicates with the abdomen in the adult, and there is ascites, it is very convenient to tap the patient through the scrotum and tunica vaginalis.

The usual quantity of fluid in hydrocele is from six to eight ounces, but Mr. Cline drew off six quarts from the celebrated Gibbon. The fluid is generally yellow, transparent, and saltish to the taste. It sometimes contains a quantity of white flaky matter produced by chronic inflammation; this is oftener observed in West Indians than in others; but Sir Astley has seen it in English patients. When produced under acute inflammation of the testis, the fluid is sometimes of a red colour, from a mixture of the red particles of the blood; the same appearance is observed when the hydrocele has immediately followed a blow.

Sir Astley has seen in the fluid of hydrocele loose cartilaginous and osseous bodies. They are accompanied with some inflammation of the tunica vaginalis; they grow by stalks from the surface of the testis and extremity of the epididymis, and, becoming detached, are found loose in the tunica vaginalis. When hydrocele has long existed, the tunica vaginalis becomes thickened like parchment, and opaque. It is also occasionally ossified; a product of chronic inflammation, and usually attended with some adhesion of the tunica vaginalis.

*Diagnosis of Hydrocele.* Transparency is in most cases a sure diagnostic mark. Its distinct and extended fluctuation, and its commencement at the lower part of the tunica vaginalis, and gradual extension upwards are also characteristic. It is less heavy than the diseased testis. The latter is more flat on the sides; more solid; painful when squeezed; the epididymis often forms a distinct swelling; the cord may be traced with ease; there is great vascularity of the scrotum; pain is generally felt in the loins; and there is usually the appearance of loss of health.

Hydrocele may be distinguished from hernia, by the occasional return of the hernial contents into the abdomen—by the dilatation of hernia in coughing—by hernia coming down from the abdomen, and by hydrocele growing upwards.

Hydrocele may be distinguished from varicocele by the latter disappearing in the recumbent posture. It is not so easy to distinguish it from hæmatocele, but the latter is usually caused by a blow, and is more solid than hydrocele. In all cases of doubt the tunica vaginalis should be punctured with a lancet.

*Causes of Hydrocele.* Dropsy, generally. Sir Astley believes that true dropsy is very rarely produced by diminished absorption. It is generally the result of an increased secretion from the arteries, which is proved by the greater vascularity of the membranous surface producing it, the thickening and other changes observed in long continued dropsies and hydroceles, and the quickness with which hydrocele succeeds inflammation of the testis and tunica vaginalis. Sir Astley allows that common hydrocele is rather the result of relaxation in the arteries and veins, than of inflammation. On injecting the absorbent vessels of the cord, they are larger on the affected than on the opposite side. Hydrocele is not unfrequently the effect of inflammation of the testis, which leaves the tunica vaginalis filled with a deep-coloured serum, often slightly reddened, and readily absorbed. Hydrocele is merely a local disease, though sometimes connected with a general hydropic tendency.

*Natural Cure of Hydrocele.* If a hydrocele be suffered to grow large and the patient be obliged to labour, excessive distention will produce inflammation of the tunica vaginalis and scrotum. A slough is formed, it separates, the water escapes, suppuration follows, granulations arise, and thus the patient is cured. Sir Astley saw this process on one occasion, and the symptoms were so severe that an unhealthy person would probably have sunk under them. Hydrocele is not always cured by a blow which tears the tunica vaginalis. In one gentleman the hydrocele disappeared in a few hours after the injury, but in six months was as large as before.

*Cure of Hydrocele by Absorption.* It is generally curable by absorption in young people. For a child, Sir Astley directs a dose of calomel and rhubarb occasionally, and a suspensory bandage, kept wet with the mur. ammon. ʒij. liquor ammon. acet. ℥vj. If the fluid be not quickly absorbed the tinctura lyttæ may be added; or the tincture of iodine may be applied. When hydrocele is the result of inflammation in the adult, calomel and colocynth, and an irritating lotion to the part often answer very well. They possess little power in common hydrocele, and Sir Astley has tried continued blistering unsuccessfully.

*Tapping for Hydrocele.* When the general health forbids the operation of injection; if a patient's fears prevent him from submitting to it; or if it be inconvenient to undergo any other operation, then the water is to be removed by tapping. We require a trocar and a canula. The latter should be two inches long and the eighth of an inch in diameter. Sometimes the tunica vaginalis is opened with a lancet, an awkward proceeding and leading to bleeding and hæmatocele. Before performing the operation, the swelling should be examined by the candle and careful manipulation, to ascertain the exact situation of the testis and cord. When partial adhesions of the tunica vaginalis have been produced, the spot for the introduction of the trocar must of course be varied.

Sir Astley's mode of performing the operation consists in making the patient stand before him, whilst he grasps the scrotum and swelling firmly with his left hand, and introduces the trocar two-thirds of the swelling downwards and with a slight obliquity upwards. When the canula has entered the tunica vaginalis the trocar is withdrawn, the canula is then passed farther into the tunica vaginalis, and the water escapes. When the trocar is introduced the swelling is grasped, that the fore-part of the scrotum may be put upon the stretch. The trocar is directed slightly upwards that the testis may not be wounded. The canula is introduced further when the trocar is withdrawn, for a similar reason. When the water has been removed and the canula is withdrawn, a piece of adhesive plaster should be laid over the wound, and a suspensory bandage should be applied.

This operation rarely succeeds in curing the disease. A strong stimulating lotion should be applied immediately after it. Exercise directly afterwards occasionally produces inflammation and a cure. Sir Astley has known a patient cured, by travelling by coach to Manchester soon after the operation. Such exertions are dangerous in old and unhealthy persons. The time of re-accumulation of a hydrocele after tapping is very uncertain. If it reappear quickly it is a proof of hydropic disposition, and requires calomel and squill at night, digitalis, sp. æth. nit. and camphor mixture twice daily. That the operation is sometimes dangerous was shewn in the case of an aged gentleman, who took a long walk on the evening of the day on which it was performed. Two days afterwards inflammation came on, gangrene followed, and in a week the gentleman expired. "There are some we must not touch; and some we cannot kill." Mr. Green, of Lewisham, has published another fatal case of gangrene of the scrotum after this simple operation. The patient was elderly, rather unhealthy, and took a long walk soon after the operation.

Sir Astley cautions surgeons against performing even the slightest operations rashly, and without the most careful precautions. The state of the constitution should always form a prominent object of attention. We would add a remark to this. Let surgeons be cautious how they meddle with old persons, for in them the state of the arteries and the general feebleness of the constitution give a marked disposition to gangrene, and sloughing of the cellular membrane. We have seen an old man perish by low erysipelas, after opening the tunica vaginalis with caustic—an old man died of sloughing of the scrotum and cellular membrane of the abdomen, after the removal of a fatty tumour in the groin—old persons frequently bring on gangrene in the lower extremities by a scratch, or cutting a little deeply on a bunyon—there is a peculiar carbuncular ulcer on the legs of old persons, depending on death, or something like it, of the cellular membrane, exceedingly intractable, and occasionally ending in gangrene—we have seen a middle aged man die of sloughy abscess in the leg, and, on dissection, the femoral artery of that side was almost obstructed by ossification—in fine, not to multiply examples, let surgeons beware how they meddle in the way of operation with elderly individuals. We know that very old men have been operated on with success, but nothing, save urgent necessity, should induce surgeons to touch them with the knife.

*Operations for the Cure of Hydrocele.* These have been many, some of them severe, others uncertain in their issue. Excision of the tunica vaginalis was formerly practised, but Sir Astley hopes never to witness it again; it was unwarrantably painful in its performance and violent in its consequences, for Sir Astley once saw both scrotum and testis slough after it. Secondly—a tent was introduced into the tunica vaginalis; the adhesions were often partial and the disease was not cured. Thirdly—potassa fusa was rubbed upon the scrotum till its influence reached the tunica vaginalis. Inflammation succeeded, the slough separated, and the cavity of the tunica vaginalis was partly obliterated by adhesion, partly filled up by granulation. When well managed this was a successful operation, but then it required great attention, was occasionally very severe, and a patient of Mr. Cline died from its effects. We have mentioned a similar case. The patient was in St. George's Hospital under the care of Mr. Brodie.

The operations to which Sir Astley has occasionally recourse, are injection—seton—and incision. The object of the first is to produce adhesive inflammation or prevent further secretion; of the two latter to fill the cavity by granulations.

Injection, for which we are indebted to Sir James Earle, has certainly proved a boon to mankind. The apparatus required is an elastic gum bottle, fitted with a brass cylinder to receive a stop-cock, which can be attached at pleasure; a trocar; and a canula two inches long. The usual injection consists of equal parts of port wine and water, or, if that has failed, two-thirds of wine and one of water. As port wine varies exceedingly in its strength, Sir Astley recommends the use of zinc. sulph., one drachm to a pint of water. One part of sp. vin. to five of water, has also been employed; and cold water often succeeds, but has also failed. The serum drawn from the hydrocele has been injected, but Sir Astley does not much like the choice. Milk was once thrown in by himself; it returned curdled, some of the larger portions remained in the tunica vaginalis, and suppuration was produced. We proceed to Sir Astley's method of performing the operation.

"The patient is to be placed in a recumbent posture upon a sofa or chair, and the surgeon to sit by his side. The tumour is grasped in the surgeon's left hand, so as to render the scrotum tense, and the trocar is to be thrust in gradually and obliquely: it should enter one-third from the lower part of the swelling, and be directed, not immediately downwards to the testicle, but a little obliquely upwards. The trocar and canula having entered the tunica vaginalis, the trocar is to be withdrawn; and in doing this the surgeon should not only nip the scrotum, but the tunica vaginalis around the canula, to confine it within the bag; and when the trocar is withdrawn, he is to push the canula to its hilt within the tunic, and the water then escapes into a basin provided for the purpose. The surgeon, putting the stop-cock on the elastic bottle, introduces the stop-cock into the canula, and the contents of the bottle are then thrown into the tunica vaginalis, great care being taken to nip the tunic upon the canula. The patient first feels pain in his groin, next near the spinous process of the ilium, and then in the loins; sometimes he complains of uneasiness at the neck of the bladder. The fluid is to be withdrawn at the end of five minutes, and the operation is thus completed." 186.

Sir Astley retains the injection within the scrotum for five minutes in adults, unless the pain be absolutely intolerable. As the succeeding inflammation is not by any means commensurate with the suffering at the time, the injection should not be withdrawn except under urgent circumstances. In young persons three minutes are sufficient. Sir Astley always throws in less fluid than he had withdrawn, and moves it about in the tunica vaginalis, to apply it to every part of the surface. If much be injected the cremaster contracts, part is forced by the side of the canula into the cellular membrane of the scrotum, and sometimes inflammation and sloughing are the consequence. If he finds the testis somewhat enlarged, Sir Astley still proceeds with the operation, for the enlargement is sometimes diminished by it, and does not prevent its success. Much depends on the treatment after the operation. The suspensory bandage is to be forbidden. "If," says Sir Astley to the patient, "you are in much pain, lie down, eat little and drink only diluents—if you suffer little, take exercise, your dinner, and two or three glasses of wine. Come to me to-morrow." If then there be redness of the scrotum, considerable tenderness, and some swelling, the suspensory bandage is to be worn, the exercise moderated, the diet to be light. If there be little appearance of inflammation, grasp the scrotum in one hand, and gently tap it a few times with the other so as to produce slight pain. Exercise and good diet are to be recommended, until the inflammatory swelling be nearly as great as the previous enlargement from the hydrocele. The swelling increases for a week, is stationary for a few days, then declines and in three weeks has subsided. The operation seldom requires more than a few hours' confinement, sometimes a week; patients are generally able to follow their occupations after four days. Sir James Earle said that he scarcely ever failed; Sir Astley sometimes does so, and would more frequently, were it not for his care in the after-treatment. Sometimes when water is reproduced in a few days after the operation, he removes it by tapping, in order to produce a larger share of inflammation.

Sir Astley has seen suppuration after injection in very irritable persons, and in cases where hydrocele was the result of inflammation not completely subdued before the injection. It has occasioned confinement and delay, and made the operation more painful, but likewise more certain. Sir Astley performed injection on one side, for a young man with a hydrocele on both, and he walked a distance of two miles home. Suppuration followed. Sir Astley injected the other hydrocele and made the patient keep his bed, yet again suppuration occurred. When cysts grow between the tunica vaginalis and albuginea, the operation will necessarily fail occasionally. If the tunica vaginalis be previously divided into different cavities by adhesion, the operation will affect no more than the particular bag injected. Sometimes the injection is thrown into the cellular membrane of the scrotum, and extensive sloughs, nay, even death, are the consequence. A dresser at Guy's Hospital obtained the permission of the surgeon to inject a hydrocele. He threw in the fluid with great difficulty, the man complained violently, the injection could not be withdrawn, inflammation and gangrene ensued, and the patient died in a week. The canula had not passed into the tunica vaginalis. If the canula does enter it, but is not confined in it by pinching the tunic around it, the same disastrous consequences will follow, as Sir Astley has often had occasion to observe.

The cure is generally effected by the effusion of serum and fibrine into the tunica vaginalis. The serum becomes absorbed, the fibrine glues the sides of the tunic together, and at length it is in great degree absorbed also. Mr. Headington dissected a hydrocele that had been injected, and the tunic adhered. It is in cases in which it does not adhere, that the disease is apt to return. This effusion, however, is not necessary to the cure, which sometimes appears to be produced by a change of action in the vessels. Sir Astley cured a hydrocele by injection. Some years afterwards the patient died, and on dissection it was found that the tunic had adhered very partially; it was more relaxed than usual, but contained no water.

*Operation by Incision.* When obscurity hangs over the nature of the case with respect to its connexion with hernia, or with some disease of the testis, it is occasionally necessary to open the tunica vaginalis. This was one of the earliest operations for hydrocele, but it often failed, in consequence of the two surfaces of the tunica vaginalis not coalescing. To obviate this uncertainty, Mr. Hunter introduced some extraneous body within the cavity. He began an incision at the upper part of the swelling and extended it two-thirds downwards, for, if lower, the testis is liable to exposure and inflammation. The water then being discharged and the state of the testis ascertained, a little flour was sprinkled in the wound, and thus the surface was forced to granulate. The operation was very certain, but very severe. It should only be resorted to in cases of great doubt concerning the state of the testis. In one case Mr. Chandler introduced lint, instead of flour, into the tunica vaginalis. Great constitutional irritation followed and the patient sank. After the operation a poultice only should be applied. Some have excised a portion of the tunica vaginalis, but this operation has proved uncertain, and is not now performed in England.

*Operation by the Seton.* When hydrocele in young children will not yield to stimulating lotions, Sir Astley passes a common curved needle and thread transversely through the middle of the hydrocele, including about an inch and a half of integument and one inch of tunica vaginalis. He then ties the thread in a knot, leaving it hanging loosely in the tunica vaginalis and scrotum. No confinement is necessary. In about a week inflammation is established, and then Sir Astley withdraws the thread, when the adhesive inflammation produces the cure. Sir Astley occasionally performs the same operation in the adult, when injection has not occasioned sufficient inflammation, and before the latter has entirely subsided.*

### V. HYDROCELE OF THE SPERMATIC CORD.

This disease is an accumulation of fluid in the sheath of the spermatic cord. It is rather rare: When the testis descends from the abdomen, the cord is closely invested by peritoneum which adheres to its vessels. The portion of peritoneum which descends before the testis from the lower part of the abdomen, does not at first adhere to that which is closely united to the cord, but a channel admitting of a probe is left between them. After a time there is adhesion of the tunica vaginalis, from the internal ring nearly to the testis. Sometimes it happens that in parts of the cord the adhesion is incomplete, and then a space is left in which a slight secretion proceeds; if this be increased, a hydrocele of the cord at this part is the consequence. A similar swelling may be produced by an accumulation of fluid in a cyst in the cord.

This swelling, when seated below the ring, is easily distinguished. It is

---

* We may here introduce the substance of a letter from Mr. Caddell to Sir Astley Cooper, on hydrocele as it occurs in the West Indies. It is found in the Appendix to the present work.

"Hydrocele is a very frequent disease in Barbadoes, but not the every-day disease that perhaps you have been led to believe. I think the cellular membrane of the scrotum is generally more thickened than in hydrocele here. I think the epididymis and cord are much more apt to be varicose or thickened. Persons in general suffer their hydroceles to grow larger, before seeking relief from them. Injection is as successful in Barbadoes as in England. It was a matter of indifference to me whether I used Solution of White Vitriol, or diluted Port Wine. I have injected hydroceles of three pints successfully; but beyond that size, and even for that size, I preferred Caustic.

Wherever the fluid varied *very much* from the ordinary fluid of hydrocele on first drawing off, and collected again, or where the tunica vaginalis has been filled with blood after the first drawing off, I have used Caustic. I have lost a few patients from erysipelas—a few from tetanus: the latter I believe is a danger unknown here; but in Barbadoes it occurs often enough to make a man avoid operations of every kind as much as he can. Cancer and pulpy testis are the ordinary diseases of the testis; but not more common than in England. Elephantiasis affects the scrotum. I have relieved the scrotum when immensely large, by pressure with adhesive plaister."

globular, of a light blue colour when grasped and raised, very transparent, firm to the feel, unattended with pain, rarely of considerable size, and merely an inconvenience to the patient from the impression it produces on his mind. When seated in the inguinal canal, above the ring, the diagnosis is very difficult. Like hernia, it disappears under pressure, is very apparent in the erect and almost gone in the recumbent posture; but there is no pain, no gurgling, no interruption to the bowels in consequence of the tumour.* The disease feels like a bullet lodged in the cord. If left to itself it increases, and at last emerges from the ring, when its transparency decides its nature. On dissection the bag is found covered by the cremaster, and the water is contained between the two portions of the tunica vaginalis of the cord, which are greatly thickened, and have no communication with the cavity of the tunica vaginalis of the testis.

In the treatment of this disease injection is difficult, and the complaint is apt to return. In a case in which injection twice failed, Sir Astley succeeded by making an incision and introducing flour; two abscesses formed during the cure. In a case at present in St. George's Hospital, a cure has been nearly effected by making an incision, and dressing in with lint. By the way, it is curious that no less than four cases of hydrocele of the cord have occurred at that hospital within this month. In one case the patient, a boy, declared that it appeared suddenly after being thrown from a cart. By rest and the ammon. mur. lotion it disappeared, but returned on the patient's resuming exercise. We believe that it was a second time dispersed by the means which answered at first. A seton, made by introducing a common curved needle carrying a single silk, is a very lenient cure. Sir Astley is of opinion that a hydrocele sometimes forms on the cord from a secretion proceeding into a hernial sac, shut at its orifice to the abdomen; the fluid is then colourless. Sir Astley inserts an amusing case, to show the difficulties of diagnosis; this will be noticed in another place.

### VI. INFLAMMATION OF THE TUNICA VAGINALIS.

By inflammation, effusion is produced into the tunic; if it proceed, fibrine mixed with serum is poured in, and the two portions of the tunic coalesce. If the inflammation be immediately subdued, the effused matter is absorbed in part or in whole; but if it continue for some time, the adhesion remains, and produces many of those indurations perceived after inflammation of the testis and its tunics. If the posterior or lower part of the epididymis have a knot in it, it has arisen from inflammatory condensation of the cellular tissue uniting the convolutions of the vas deferens. If a hardness is felt on the surface of the testis, it indicates the existence of partial adhesion and thickening of the tunica vaginalis. In one case Sir Astley found, in addition to these, adhesive matter poured into the substance of the testis in two situations, the larger part at the rete. When the two portions of the tunic adhere, injection passes through the new matter,

* We have remarked on a former occasion, that this diagnostic mark is to be received with the understanding, that symptoms of intestinal obstruction or inflammation may be accidentally combined with the tumour. Such a circumstance has occurred more than once within these few years.—*Rev.*

which is organized. The only bad consequence of these adhesions is, that the testis does not slip so readily out of the way of injury or violence.

The treatment of acute inflammation of the tunica vaginalis is the recumbent posture, support of the part, leeches, and evaporating lotions. The hydrocele following it often disappears under the use of stimulating lotions. Suppuration of the tunica vaginalis is very rare, except as a consequence of hydrocele, and its treatment. It is, however, the natural cure of hydrocele, when the bag is excessively distended; for suppuration and ulceration ensue, the water is discharged, and the cavity fills up by granulations.

Chronic inflammation of the tunica vaginalis, producing an effusion of water into its cavity, is seen in the inhabitants of warm climates, and more especially in those of the West Indies. The same effect is produced in persons subject to disease in the urethra and prostate; and indurations, apparently of the epididymis and testis, but often in reality of the tunics only, with small collections of water, ensue. In performing operations for such hydroceles, Sir Astley has found the tunica vaginalis so extremely thickened that, when opened, it has remained separated from the testis, and has been as stiff and thick as wetted parchment. The contained fluid is sometimes of a white appearance and opaque; if suffered to remain in the vessel into which it has been drawn, it precipitates numerous white flakes of adhesive matter, whilst the water above it is clear and colourless.

It is chiefly chronic inflammation of the tunica vaginalis which leads to the variety in the situation of the water in hydrocele, and to the danger of wounding the testis with the trocar. In these cases cysts are formed in various directions, and the testis therefore loses its usual situation with respect to the effusion. In these cases also incision is occasionally required, when obscurity hangs over them from opacity of the tunics, and when more than one bag containing water has been formed.

### VII. Cartilaginous Bodies in the Tunica Vaginalis.

In dissecting persons labouring under hydrocele, we find little loose bodies floating in the serum. They are sometimes numerous, more frequently two or three in number, sometimes only one. They appear at first sight like cartilage; on more attentive examination they are found to be cartilaginous on their surface, but ossific internally. In this respect they resemble the bodies found loose and pendulous in joints. In some cases Sir Astley was unable to discover from whence they had been formed, although there were marks of inflammation and adhesion in the tunica vaginalis.

In other cases our author has seen them produced in two ways. First.—They formed pendulous bodies, hanging from the epididymis and testis, covered by the tunica vaginalis; when the stalk becomes small, they fall or are broken off by the motions of the testis and the pressure to which they are exposed. Of this variety Sir Astley has given a plate. Secondly,—They are produced upon the surface of the testis, between the tunica vaginalis and albuginea, in a cyst which, when opened, contains one of these little solid bodies, pendulous from its internal surface. The rationale is not difficult. Chronic inflammation produces an effusion between the tunica vaginalis and albuginea; it projects and is pendulous; becomes vascular from the tunics; and in it, cartilage is first, and ossific matter afterwards secreted. It is a curious circumstance that all the serous and sero-fibrous membranes have a disposition to form cartilaginous and earthy matter. Probably it depends on the general fibrous character of their inflammatory effusions. Sir Astley has never been able to detect the existence of these substances in the living body.

### VIII. OSSIFICATION OF THE TUNICA VAGINALIS.

This is rare. When found, it is usually the concomitant of old hydrocele, for Sir Astley has only seen one case in which they did not exist together. The earthy matter is thrown out in patches of various size; in one preparation at Guy's Hospital, it has been deposited in the form of numerous spiculæ.

A portion of the ossific matter was sent for analysis, from Sir Astley to Mr. Barry, of Lombard-street. When divested of membrane, and dried, 100 parts consisted of—phosphate of lime 45—carbonate of lime, with a trace of magnesia, 17—animal matter, 38.

The tunica albuginea undergoes a similar change of structure. These deposites are the consequence, no doubt, of chronic inflammation. Of this, diseases in the urethra are the most frequent cause, and attention to the state of that canal is the principle to guide our treatment.

### IX. FUNGOID INFLAMMATION OF THE TUNICA VAGINALIS.

The only information offered by Sir Astley on this subject is in the shape of a case attended by Mr. Brodie and himself. The disease must be extremely rare.

*Case.* Mr. T., æt. 60, rather bloated and unhealthy, had for fifteen months observed a swelling on the left side of the scrotum, unattended with pain, but accompanied by water in the tunica vaginalis. Mr. Brodie employed the mercurial treatment, leeches, and the recumbent posture with little effect. The tunica vaginalis was then punctured, and about two ounces of fluid drawn off; the treatment was still pursued; the water was removed a second time; but still no improvement was perceived. Sir Astley was consulted and recommended perseverance in the same mode of treatment. After a lapse of several weeks, Sir Astley and Mr. Brodie agreed to remove the part, if, on puncturing, the fluid should be found to be inconsiderable. A lancet was passed into three different parts where fluctuation seemed distinct, but no fluid issued. Mr. Brodie then removed the testis.

On dissection the testis was perfectly sound. The vas deferens could only be injected to the beginning of the epididymis, which was drawn to a great length by the swelling and terminated in a membranous cord. The cavity of the tunica vaginalis was occupied by a spongy effusion, having all the character of incipient fungus. The tunica vaginalis was thickened and had a large piece of ossific matter in it. The cord was unaffected.

The patient died of erysipelas, and on examination his body was found in other respects free from disease.

### X. On Hæmatocele.

Hæmatocele is a collection of blood in the tunica vaginalis testis. The swelling is pyriform like hydrocele, not painful, does not affect the general health, is attended with slight fluctuation, is not at all transparent. It is distinguished from hydrocele by its greater weight, its want of transparency, its obscure fluctuation, and its being usually the consequence of a blow. On dissection, the tunica vaginalis is found excessively thickened. The blood, which is coagulated, is coffee-coloured, and if a fluid be contained with it, that assumes the same appearance. The blood in the tunica vaginalis is found in three conditions according to circumstances. First—it is entirely coagulated. Secondly—some fluid blood is found with the coagulum, when the swelling is extremely tense, and, if opened, bleeding is apt to recur from the orifice of the vessel, after the pressure is removed. Thirdly—when the disease has been accompanied by inflammation a serous fluid is secreted.

*Case.* Mr. N., was brought to Sir Astley, with a pyriform swelling of the scrotum, as large as a double fist. The testis and epididymis could be felt at the lower part of the swelling, and above it, as high as the ring, a solid substance united with a fluid was perceived. It was not transparent, and occasioned no inconvenience, excepting from its size and weight. It had existed seventeen years, and was attributed to a blow from the pommel of the saddle, received in hunting. Sir Astley opened the swelling at his house, and discharged a coffee-coloured fluid blood, with a solid substance of a brownish yellow colour. The tunica vaginalis was as thick as the thickest parchment. Sir Astley introduced a piece of cotton wool, and the patient went three miles to his home, in a coach. On the same day, whilst sitting in his counting-house, he was seized with a profuse hæmorrhage from the tunica vaginalis, and fainted. Violent constitutional irritation followed, with suppuration of the tunica vaginalis. Warm poultices and the magnes. sulph. with liq. ammon. acet. were employed. About the third or fourth day large clots of blood came away, and were followed by profuse discharges of pus, when the febrile symptoms subsided. The discharge continued for some time, then ceased, and in six weeks after the operation the patient was perfectly well.

Hæmatocele now and then follows tapping in hydrocele, especially if a lancet be employed. A surgeon tapped a patient twice for hydrocele. Two months after the performance of the last operation, he returned with the swelling somewhat rounder than before. It was again tapped and a pint of thick bloody fluid drawn off. In a fortnight the swelling reappeared, but is gradually subsiding.

Hæmatocele is often connected with hydrocele, and is a consequence of it. A man received a severe blow upon an old hydrocele, which bruised the scrotum, made the swelling increase suddenly, and occasioned great pain. Sir Astley made an incision into it, and discharged a large quantity of water and coagulated blood. There was a rent one or two inches in length in the tunica vaginalis, covered with coagulum. Dr. Saunders had a hydrocele which was occasionally tapped. He struck the scrotum against the back of a chair, and the hydrocele, as he imagined, returned. Mr. Lucas tapped it, but no water passed. Mr. Cline made an incision into the tunica vaginalis, which was found full of blood; a poultice was applied, and the Doctor soon recovered. Sir Astley mentions another case, but this we need not detail here.

Hæmatocele is not always produced by a blow. Sir Astley attended a gentleman with a large pyriform swelling in the left tunica vaginalis, which had never been painful, had obscure fluctuation, and was not transparent. Sir Astley made an incision into the tunica vaginalis, and discharged nearly a pint of fluid blood. This swelling had not succeeded a blow, but the gentleman had been in the habit of making great exertions.

In the collection at St. Thomas's Hospital is a hæmatocele, for which the testis was removed by mistake. The surgeon who removed it had not even the curiosity to examine it, but when Sir Astley cut into it, he found the tunica vaginalis extremely thickened, and filled with coagulated blood of a brownish red colour. The testicle was placed at the posterior and inferior part of the swelling.

*Treatment.* This must vary according to the manner in which the disease is produced. If occasioned by a blow and unaccompanied by hydrocele, the patient must observe the recumbent posture, apply leeches, keep the bowels open, and, if there be much pain, we must even take away blood from the arm. The liq. ammon. acet. and sp. vini ought to be applied to the part. If connected with hydrocele, unless there be much inflammation, the best plan of treatment consists in making an incision into the part, discharging the contents of the tunica vaginalis, and, without introducing any foreign body, leaving the case to the natural process of inflammation. When it occurs without any apparent cause there is something wrong in the constitution, requiring attention as well as the local disease. There is generally visceral disease, especially in the liver, sometimes obstruction to the circulation through the chest. When this has been remedied by medicine, an incision may be made into the tunica vaginalis. The recumbent posture is in this case very essential in preventing bleeding after the operation. In hæmatocele it is particularly necessary to cut into the tunica vaginalis, to examine the state of the testis, as castration has often been performed when the latter was perfectly sound.

Excessive irritation sometimes follows the operation. A gentleman had a hydrocele on both sides. The water was drawn off from the right side by a lancet, but in a few days the swelling was larger and harder than ever. Two years afterwards he was seen by Sir Astley. The swelling on the right side was not transparent; on the left it was. Sir Astley suspected hæmatocele, and punctured the tunica vaginalis, when fluid blood of a coffee-ground appearance was discharged. The tunica vaginalis was extremely thickened, but the testis appeared to be sound. Dangerous irritation followed, but the patient ultimately recovered.

We have seen the most severe irritation produced by the operation for hæmatocele, but the patient was finally cured. A curious case happened lately to Mr. Hawkins at St. George's Hospital. A man had long suffered from hydrocele on the left side, which had frequently been tapped. In walking he squeezed the testis between his thighs, a sudden swelling took place, and he fainted; the tunica vaginalis had given way, and the hydrocele had got abroad into the neighbouring cellular membrane. Great ecchymosis of the scrotum, thighs, and hypogastrium followed. The rent in the tunica vaginalis healed, and Mr. Hawkins introduced a trocar; the opening was instantly blocked up with coagulum. Irritation and fever followed. The tunica vaginalis was laid open by incision, and much coagulated blood with coffee-coloured fluid found in it. It was dressed in with lint —some sloughing succeeded—then suppuration—and, ultimately, the patient was discharged, with only a small sanious opening in front of the scrotum. The cord remained much thickened—the testicle, though adherent to the scrotum anteriorly, was sound.

### XI. On Varicocele.

Various causes conspire to produce more distention of the spermatic veins at one time than another. Among these may be enumerated the erect position; relaxation of the body from heat or weakness, for which reason the inhabitants of warm climates are very subject to varicocele; corpulency, in which the fat of the mesentery and omentum prevents the free return of blood in the veins; pressure from dress, or a belt round the abdomen. It occurs more frequently on the left side than the right, for two reasons. In the first place, the spermatic vein of the left side, instead of ending in the vena cava like the right, terminates in the left emulgent, nearly at right angles with the stream of blood from the kidney. In the second place, the left testicle usually hangs lower than the right, and hence the veins are somewhat larger, and the height of the column of blood is greater. Whatever be the cause, the diameter of the veins on the left side is increased, they become more tortuous, and their coats are thickened.

Varicocele can scarcely be termed a disease, for in the majority of instances there is neither pain, inconvenience, nor diminution of the virile powers. A gentleman of Sir Astley's acquaintance had a slight hydrocele on one side and a varicocele on the other, yet he married and had a numerous offspring. In a few cases it produces uneasiness in the loins and in the course of the cord. The patient's mind, however, is distressed by it. He thinks he has a serious, or an effeminating disease, and consults one surgeon after another, till time and experience teach him his error. Sir Astley has now and then seen the disease on the right side only, very rarely on both.

*Dissection.* All the veins are enlarged, and so much elongated, that the vein in the course of the vas deferens reaches much lower than the testicle itself, which is thus seated on its fore part, whilst much of the varicocele lies below it. The coats of the veins are also thickened.

*Diagnosis.* Varicocele may be confounded with hernia. Like hernia, it fills from the abdomen and descends; increases in the erect, and almost disappears in the recumbent posture; and if large, becomes distended when the patient coughs, though the latter symptom is not usually very distinct. Unlike hernia, it feels like a bag of worms in the scrotum, between which the fingers will sink and meet, instead of being a smooth and uniform swelling; it is also unattended with interruptions of the bowels, and with the gurgling of intestinal hernia. The main and convincing point of distinction is this. The patient being recumbent and the testicle elevated till the veins are emptied, the surgeon places his finger at the abdominal ring, and the patient is directed to rise; all hernial descent is of course prevented, but the varicocele again appears, in consequence of the blood getting into the veins through the spermatic arteries. This will even take place if the patient does not rise at all. Notwithstanding the simplicity of the diagnosis, Sir Astley has again and again seen trusses applied for this disease, with the most pernicious effects.

*Treatment.* The indications are, to prevent the increase of the varicocele, and remove the existing inconvenience. First—The part should be supported in a silk-net suspensory bandage, to lessen the column and weight of blood. Secondly—The clothes should be thin and light, to keep the part as cool as possible. Thirdly—All pressure from the clothes on the abdomen should be prevented. General cold bathing is useful, and the patient should bathe the part night and morning with cold water, in which a mixture of nitre and muriate of ammonia are dissolving. If the veins be very large, blisters applied to the scrotum are useful. But in general it is best to relieve the patient's mind of all uneasiness and chagrin, and only recommend the mild measures enumerated above. Probably one person in every twenty has varicocele in some degree.

It occasionally happens that persons suffer great pain in the testis and loins, from a varicocele, and then it has been proposed to cut down on the spermatic vein and place a ligature upon it. Sir Astley dreads such an operation, for on several occasions he has seen death ensue from meddling with the veins of the extremities. In cases of very large varicocele, accompanied with pain, the removal of a portion of the scrotum would lead to a diminution of the veins of the cord, and might be advantageously employed. It is even occasionally necessary to remove the testis for varicocele.

*Case.* T. H. aged 18, was admitted into Guy's Hospital with a large varicocele on the left side, attended with considerable pain, referred chiefly to the loins. The testicle was soft, smaller than the other, and painful on being handled. The disease had originated three years previously, from a blow on the pommel of a saddle, which bruised the testis, and produced for a few minutes excruciating pain. He had suffered little inconvenience from the varicocele, till within the six months preceding his admission. At the patient's earnest and decided request, castration was performed, after the employment of local sedatives, leeches, the horizontal posture, and alteratives had been tried in vain.

The last chapter in this admirable work is on the

### XII. CHIMNEY SWEEPER'S CANCER.

The soot harbouring in the wrinkles of the skin, where the cuticle is thin and the cutis highly vascular, produces irritation and ulceration so much resembling cancer, as to have received that appellation. It certainly bears a strong resemblance in many points to cancer of the lip. Sir Astley has only seen it on two parts of the body, the scrotum and the cheek. On the former it is frequent, but Sir Astley has witnessed it but twice upon the latter. In one case, the sore was seated in the centre of the cheek in a very old man, the wrinkles of age upon his face having formed a nidus for the soot. In the second individual, the disease began on the cheek near the angle of the mouth, and extended both to the upper and under lip. Mr. Keate informed Sir Astley that he had known an instance of the disease in the cheek. Within these two months there have been, in St. George's Hospital, two instances of chimney-sweeper's cancer, commencing in the groin. One patient was an adult, under the care of Mr. Babington; the other a boy, now under the charge of Mr. Brodie.

The first appearance of the disease is a wart upon the scrotum, which seems broken on its surface. On this an incrustation forms. If this be rubbed away or picked off, the papillæ on the surface of the wart appear excoriated, red, and broken, and give issue to a slight discharge. An incrustation forms again upon the wart, and remains until accident or ulceration remove it, when the surface is highly vascular, and discharges a bloody serum. Still the surface becomes incrusted, till at length an ulcer of some extent forms under it. The sore is hard; its edges are indurated and everted; its surface is unequally vascular, so that it is yellow in some parts, red in others; and it discharges a bloody serum, often of offensive smell, though a purulent discharge is sometimes observed. We have seen the origin and progress of the disease somewhat different from this. It began in a small lump, not warty, beneath the skin; this enlarged; was poulticed, and opened with a lancet; blood only was discharged; an ulcer formed; and then it assumed the characters described by Sir Astley. Such at least was the history given by the patient in St George's Hospital, to whom we have alluded. It must be recollected that in those individuals the groin, and not the scrotum, was affected, and there was much surrounding glandular enlargement.

At first the patient rather complains of a troublesome itching than of pain, but, as the disease advances, darting pinching pain, with heat, shoots through the part, as in most diseases of specific nature. The ulcer proceeds, reaches the tunica vaginalis, hardens it, and affects the surface of the testis. When ulceration has become extensive, one or more of the absorbent glands in the groin grows hard and swollen: it inflames, is poulticed and fomented, breaks and discharges some pus with a bloody serum. The glands slowly ulcerate with a hardened surface and everted edges, discharging serum from some parts, a curd-like matter or white sloughs from others. At length ulceration has formed a deep chasm in the groin, or an extensive sore. If the former, the femoral vessels, or a large branch of them, are exposed and ulcerate, and life is lost by a sudden hæmorrhage; if the latter, the patient is slowly exhausted by irritation and discharge. The scrotum is in some cases nearly destroyed by ulceration. If the parts are removed by operation and dissected, it will be found that the surface of the cellular membrane surrounding the ulcer is filled with a white scirrhous substance, imperfectly vascular, and presenting much the same appearance as the cancer of the lip. The glands in the groin are enlarged and hardened—they contain a white solid in some parts, a soft white curd-like matter in others—here they are highly vascular, there they have no vascularity whatever. Sir Astley has known the disease occur at various ages, from 26 to 80 years. Mr. Brodie's patient, at present in St. George's, is a boy.

"From the number of persons who pursue this occupation, and the comparatively few affected by the application of soot, it would appear that there is something, either in the constitution or the parts of some individuals, which disposes to its production. I am inclined to believe that it depends more upon local circumstances than upon the constitution, because the subjects of it appear to be very healthy at the dawn of the disease, although they lose that health in the progress of the complaint. Some of them are, it is true, very intemperate, and are thus rendered irritable. But I believe that it is a thin cuticle, and an irritable skin, which permit the soot to produce its peculiar irritation and effusion; and that it spreads both by simple extension of surface, and by the irritation of the absorbent glands." 229.

No medicines that have yet been tried possess any power over this disease; neither can we heal the sore by local applications. Only two modes of procedure remain—to destroy it by sloughing, or remove it by excision.

A drachm of the oxyd of arsenic, well mixed with one ounce of the ceratum cetacei, is to be thickly spread upon lint, and applied to the sore for twelve hours; it is then to be succeeded by a poultice. A slough is produced by the arsenic, which separates in a few days, and leaves a healthy surface; but if any of the malignant leaven remain, the arsenic must again be applied, until an entirely new surface be produced. If the glands are enlarged and hardened, the caustic will do them harm and ought not to be applied. To remove the disease by the knife is a simple piece of dissection. Enlarged glands in the groin must not positively forbid the operation, for sometimes they are affected by irritation only, and, as excision of part of the scrotum is neither very difficult nor attended with danger, the patient should be offered the chance which it affords. If the tunica vaginalis is implicated in the disease it will require great caution in its removal, to avoid injuring the testis.

After the removal of the diseased scrotum, the vessels, though small, are apt to bleed freely, and the hæmorrhage is not readily stopped by pressure. Every vessel which bleeds should be tied, no dressings ought to be applied to the part, the patient should be kept cool and in the recumbent posture; the scrotum will then contract. When the bleeding has ceased, the edges of the wound may be brought together by sutures.

Sir Astley concludes by referring to a paper on this subject by Mr. Earle, in the Medico-Chirurgical Transactions.

We have now concluded the division of Sir Astley Cooper's work, which treats of the Diseases of the Testis. Our readers will have seen that our object has been analysis, not criticism; we may say that a closer or more careful analytical review was never offered to the medical public. In little more than fifty pages of our type, the professional reader is presented with the substance of two hundred and forty-five of the able author's volume. Every thing is condensed, but every thing is here. Like hammered metal, the value and the strength remain, though the bulk has been diminished by the labours of the smithy. Our readers may, from this analysis, perceive the absurdity of the attempt to strike off in a few pages, an effective and useful review of any valuable work. Critics may give us a short remark and a long quotation; but beyond this they cannot pretend to go, without deluding themselves or others. A medical review, to prove useful, should be chiefly analytical; and for such analysis, space, time, and labour are required. Away then with the farce of affecting to put a review in a corner; of making it a small and inconsiderable department. A notice this may be, too often an advertisement, inserted by a friend; but a review it is not.

It may be expected that, on parting with our able author, Sir Astley Cooper, we should offer a summing up. It is unnecessary. There is but one opinion on this work, there can be none other. It is one of the most valuable contributions to practical surgery, which the nineteenth century has witnessed. Every page, every line stamps the able practitioner, the acute observer, the indefatigable treasurer of facts. It is by these, that Sir Astley Cooper has risen to his present eminence;—by noting what he has seen, not by revelling in what he has imagined. Such men as Sir Astley Cooper and Mr. Brodie have not electrified the world by brilliant hypotheses, which flare for a season, dazzle men's eyes, and go out; they have studied cases closely; have noted what they saw; have become in consequence first-rate practitioners; and will, we trust, prove severally, ere the course of nature shall have consigned them to the tombs of Cline and the Hunters, benefactors by their writings to mankind.*

---

* In our last number, whilst reviewing the able work of Sir Astley Cooper on the Diseases of the Testicle, we made the following remark on the "hydatid disease" of that organ.

"We are afraid that Sir Astley has overrated the mildness and local character of this affection. In a case at St. George's Hospital, in which castration was performed by Mr. Brodie on a testicle exhibiting the hydatid disease, death from peritonitis ensued, and an opportunity was afforded of examining other parts. The lumbar glands *were* affected in a similar manner with the extirpated testis."

Since this was written we have found that we were in error. Sir Astley Cooper has kindly shewn us his preparation of the hydatid testis, and we have taken the opportunity of comparing it with that from Mr. Brodie's patient. They evidently represent different diseases. In Mr. Brodie's the cysts are few and small, and separate small cysts have fallen from their interior. The cysts, too, are deposited in a yellow fibrous sort of substance, and the structure of the testis is expanded around it.

The testis in the cabinet of Sir Astley Cooper, on the contrary, consists of innumerable cells, divided from each other by membranous partitions. They contain no loose cysts within them. There is no new fibrous or other deposit, and no vestige of the structure of the testis remains.

The differences between the two preparations are obvious, and it gives us much pleasure to acknowledge our error. We did not question the accuracy of Sir Astley Cooper; who could do so? We are happy to find that a fact which seemed to militate against his conclusions has proved confirmatory of their soundness and value. We might almost say, that we are proud of being proved in the wrong by such men as Sir Astley.—*Rev.*

